# Supplementary material for: The allosteric inhibition of glycine transporter 2 by bioactive lipid analgesics is controlled by penetration into a deep lipid cavity
Source: J Biol Chem. 2021 Jan 12;296:100282. doi: 10.1016/j.jbc.2021.100282 (PMC7949037; doi:10.1016/j.jbc.2021.100282)
Supplement: Supplemental Figures and Tables [file mmc1.pdf]

The allosteric inhibition of glycine transporter 2 by bioactive lipid analgesics is controlled by penetration into a deep lipid cavity

Supporting Information

Katie A. Wilson,[1] Shannon N. Mostyn,[2] Zachary J. Frangos,[2] Susan Shimmon,[3] Tristan Rawling,[3] Robert J. Vandenberg,[2] and Megan L. O'Mara[1]*

[1]Research School of Chemistry, College of Science, The Australian National University, Canberra, ACT, 2601, Australia

[2]Discipline of Pharmacology, School of Medical Sciences, University of Sydney, Sydney, NSW, 2006, Australia

[3]School of Mathematical and Physical Sciences, University of Technology Sydney, Sydney, NSW, 2007, Australia

Corresponding Author: Megan L O'Mara

Email: megan.omara@anu.edu.au

Running title: Bioactive lipid inhibitors of glycine transporter 2

Supporting Information

Table of Contents

Supplementary Methods 2

Table S1. Distances between the residues defining the intracellular and extracellular gates of GlyT2 over the total simulation time. 4

Table S2. Average membrane properties over the total simulation time when lipid inhibitors are bound in the extracellular allosteric binding site of GlyT2. 4

Table S3. Percentage of the total simulation time in which residues are in contact with the C18ω9 lipid inhibitors that are bound in the main channel of GlyT2. Only interactions that occur for >30% of the total simulation time are reported.[a] 5

Table S4. Percentage of the total simulation time in which residues are in contact with the Lys lipid inhibitors that are bound in the main channel of GlyT2. Only interactions that occur for >30% of the total simulation time are reported.[a] 6

Table S5. Percentage of the total simulation time in which residues are in contact with the Lys lipid inhibitors that are bound in the main channel of GlyT2. Only interactions that occur for >30% of the total simulation time are reported.[a]...7

Table S6. Hydrogen bonding interactions[a] occurring between the transmembrane helices in GlyT2 when no bioactive lipid is bound (control) or a bioactive lipid is bound for >30% of the total simulation time. 8

Figure S1. Average intermolecular distance (Å) from the end of the tail to the stereocenter of lipid inhibitors. 9

Figure S2. Positioning of key amino acids around the C18ω9 Lys lipid inhibitors bound in the extracellular allosteric binding pocket of GlyT2. 10

Figure S3. Positioning of key amino acids around the C18ω9 Trp lipid inhibitors bound in the extracellular allosteric binding pocket of GlyT2. 11

Figure S4. Positioning of key amino acids around the acyl lysine lipid inhibitors with varying tail lengths bound in the extracellular allosteric binding pocket of GlyT2. 12

Figure S5. Interactions between C14ω5 L-Lys headgroup and membrane POPC. 13

Figure S6. Positioning of key amino acids around the acyl lysine lipid inhibitors with varying tail lengths bound in the extracellular allosteric binding pocket of GlyT2. 14

Figure S7. Interactions between C14ω5 L-Lys headgroup and membrane POPC. 15

Figure S8. Positioning of key amino acids around the acyl lysine lipid inhibitors with varying double bond position bound in the extracellular allosteric binding pocket of GlyT2....16

Figure S9. Positioning of key amino acids around the acyl lysine lipid inhibitors with varying double bond position bound in the extracellular allosteric binding pocket of GlyT2....17

Figure S10. Average distance (Å) between a) EL2 (D329) and EL4 (Q541), b) EL2 (D329) and EL6 (Y705) and c) EL4 (Q541) and EL6 (Y705) over the total simulation time when the lipid inhibitors are bound in the extracellular allosteric binding site of GlyT2. Average distance in control simulation is indicated by a dashed line....18

Synthetic Data 19

Supplementary Methods

General chemistry

(*Z*)-octadec-13-enoic acid (**C18ω5COOH**), (*Z*)-hexadec-9-enoic acid (**C16ω7COOH**), (*Z*)-hexadec-13-enoic acid (**C16ω3COOH**), and (*Z*)-tetradec-9-enoic acid (**C14ω5COOH**) were synthesised following literature procedures [29]. BOC and methyl ester protected L- and D-Lysine (**Lys-BOC-Me**) and EDCI were purchased from Fluorochem (Derbyshire, United Kingdom). All other reagents and anhydrous solvents were purchased from Sigma Aldrich (Castle Hill, NSW, Australia). Reactions were monitored by thin-layer chromatography (TLC) using silica gel 60 F_{254} plates. TLC plates were visualised with potassium permanganate TLC stain. Reaction products were purified by dry column vacuum chromatography on silica gel using gradient elutions. ^{1}H and ^{13}C NMR spectra were recorded on an Agilent 500 MHz NMR. Spectra were referenced internally to residual solvent ($CDCl_3$; ^{1}H d 7.26, ^{13}C d 77.10. DMSO-d_6; ^{1}H d 2.49, ^{13}C d 39.52). High resolution mass spectra (HRMS) were recorded on an Agilent Technologies 6510 Q-TOF LCMS.

General procedure for EDCI coupling

To a solution of the fatty acid (**C_Xω$_Y$COOH**, 2.0 mmol) in anhydrous DMF (10 mL) was added hydroxybenzotriazole hydrate (2.40 mmol), and EDCI (2.80 mmol). The mixture was stirred at room temperature for 1 h, then BOC and methyl ester protected D- or L-Lysine (**Lys-BOC-Me**, 1.0 mmol) and triethylamine (6.0 mmol) were added. The reaction mixture was stirred for 18 h, then diluted with water (50 mL). The crude product was extracted with ethyl acetate (3 x 25 mL) and concentrated in vacuo. The crude product was purified on silica gel by stepwise gradient elution with chloroform/isopropanol (100:0 to 90:10). The products were isolated as waxy solids (yield = 60 - 85%).

General procedure for ester hydrolysis.

To a solution of the ester (**C_Xω$_Y$-Lys-BOC-Me,** 0.50 mmol) in ethanol (30 mL) was added 1M NaOH (10 mL), and the resulting solution was stirred at 40°C for 3 h. The reaction volume was reduced ethanol was removed under reduced pressure, and the aqueous residue was adjusted to pH 2 with 0.5M HCl. The resulting suspension was filtered, and the solid product washed with water (10 mL) and ethanol (5 mL) and dried in vacuo. The products were isolated as white solids (yield = 84 - 95%).

General procedure for BOC removal.

To a solution of the BOC-protected intermediate (**C_Xω$_Y$-Lys-BOC,** 0.30 mmol) in dichloromethane (2 mL) was added 2M HCl in diethyl ether (2 mL). The solution was stirred at room temperature for 4 hours and then concentrated in vacuo. The resulting solid was triturated with diethyl ether (3 x 10 mL) and dried in vacuo, yielding the product as light yellow powder (yield = 95 - 98%).

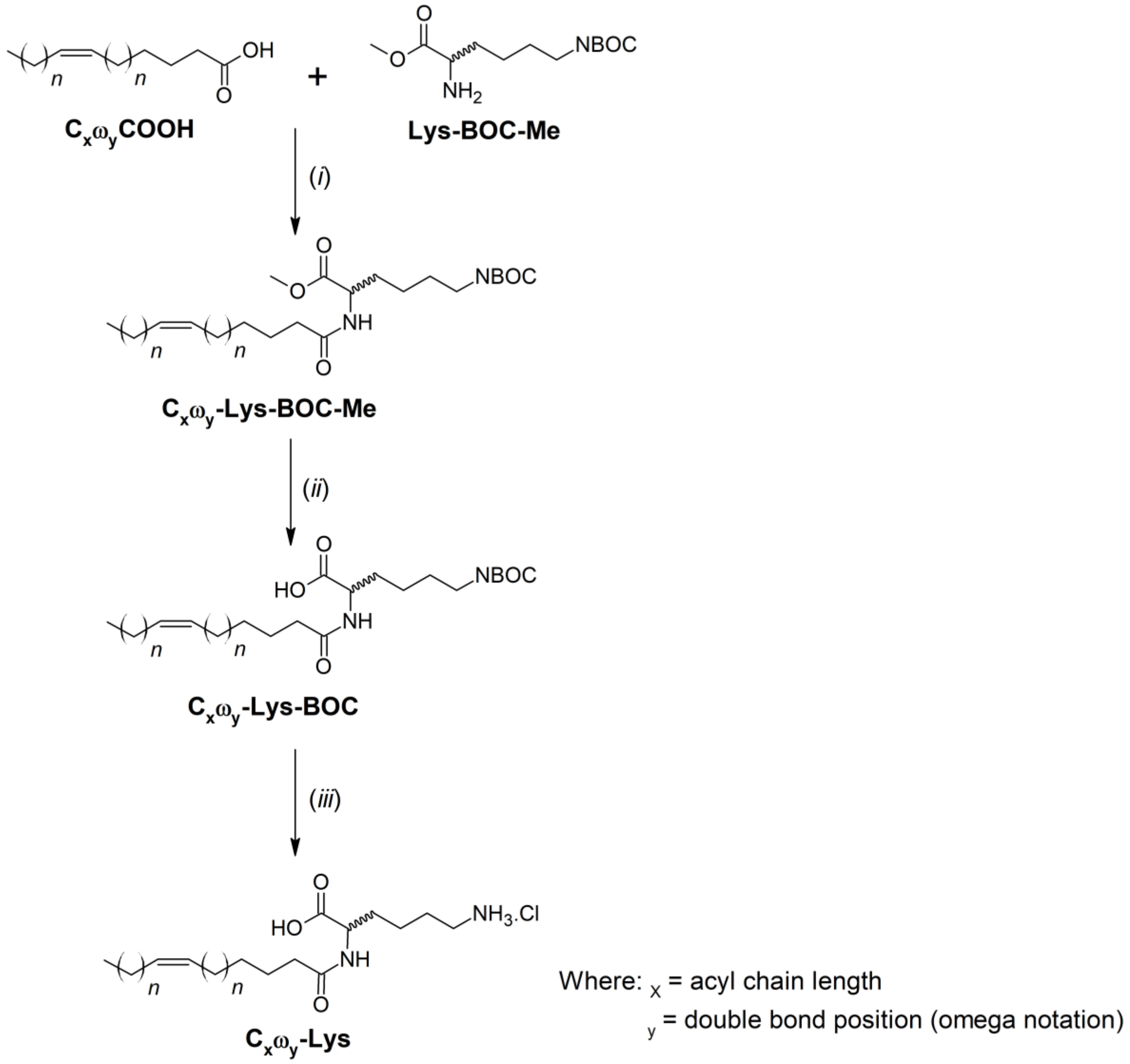

Scheme 1. Chemical synthesis of N-acyl Lysines. Reagents and conditions: (*i*) EDCI, HOBt, NEt_3, rt, 18h; (*ii*) NaOH, 40 ^oC, 3 h, then HCl; (iii) HCl, rt, 4 h.

Table S1. Distances between the residues defining the intracellular and extracellular gates of GlyT2 over the total simulation time.

	Intracellular gate (Å)	Extracellular gate (Å)
Control	8.2±0.8	5.4±0.5
L-Trp C18ω9	10.9±2.6	5.8±0.6
D-Trp C18ω9	9.6±1.6	4.7±0.4
L-Lys C18ω9	12.0±1.9	6.0±0.8
D-Lys C18ω9	8.3±1.0	5.1±0.4
L-Lys C16ω7	5.5±0.4	6.7±0.7
D-Lys C16ω7	4.9±0.3	6.8±0.6
L-Lys C14ω5	5.6±0.4	12.9±2.6
D-Lys C14ω5	5.1±0.5	9.3±1.9
L-Lys C18ω5	5.4±0.7	13.1±2.7
D-Lys C18ω5	6.1±0.7	7.3±0.7
L-Lys C16ω3	6.2±0.8	8.1±1.1
D-Lys C16ω3	4.7±0.4	10.1±2.6

[a] Intracellular gate defined as R216 and D633, and the extracellular gate is defined at R191 and D592. An inward open conformation will have an intracellular gate distance of < 4.1 Å and an extracellular gate distance of > 40.0 Å, while an outward open/occluded conformation will have an intracellular gate distance of > 4.1 Å and an extracellular gate distance of < 14.0 Å

Table S2. Average membrane properties over the total simulation time when lipid inhibitors are bound in the extracellular allosteric binding site of GlyT2.

	Thickness (Å)[a]	APL ($Å^2$)[b]
Control	42.7±0.3	55.3±1.1
L-Trp C18ω9	42.7±0.3	56.0±0.9
D-Trp C18ω9	42.8±0.4	55.7±1.1
L-Lys C18ω9	42.7±0.4	55.9±1.0
D-Lys C18ω9	42.8±0.3	55.3±1.0
L-Lys C16ω7	42.8±0.2	55.2±0.8
D-Lys C16ω7	42.9±0.2	55.3±0.8
L-Lys C14ω5	42.8±0.2	55.4±0.7
D-Lys C14ω5	42.8±0.2	55.4±0.9
L-Lys C18ω5	42.8±0.3	55.4±1.5
D-Lys C18ω5	42.7±0.4	55.9±2.0
L-Lys C16ω3	42.8±0.4	56.1±1.0
D-Lys C16ω3	42.6±0.3	56.0±1.5

[a]Membrane thickness is measured as the distance between P atoms in the POPC headgroup. [b]Area per lipid for POPC.

Table S3. Percentage of the total simulation time in which residues are in contact with the C18ω9 lipid inhibitors that are bound in the main channel of GlyT2. Only interactions that occur for >30% of the total simulation time are reported.[a]

Residue	Region	C18ω9 L-Trp	C18ω9 D-Trp	C18ω9 L-Lys	C18ω9 D-Lys
V214	TM1	44.61	69.04	71.37	32.42
W215	TM1	-	26.23	-	-
L436	TM5	86.68	89.95	61.12	85.22
R439	TM5	99.07	97.00	78.89	92.81
G440	TM5	40.21	54.06	-	39.68
L443	TM5	59.12	79.16	35.22	88.08
V519	TM7	44.81	50.4	51.8	43.68
I520	TM7	48.14	66.58	60.99	20.91
S522	TM7	-	-	76.56	-
V523	TM7	88.42	92.74	91.08	61.72
F526	TM7	92.28	94.07	93.14	98.47
M527	TM7	69.57	67.18	62.32	-
I545	EL4	34.95	33.02	44.07	-
V549	EL4	63.32	42.74	54.46	-
Y550	EL4	97.87	99.07	76.76	82.69
A553	EL4	84.62	87.08	76.56	60.92
L554	EL4	37.28	65.45	48.40	70.51
R556	EL4	52.53	43.87	22.24	63.25
L557	EL4	73.57	90.48	76.36	78.56
W563	TM8	-	35.15	34.02	49.33
F567	TM8	79.69	93.54	96.54	78.83
M570	TM8	33.42	42.54	36.15	39.55

[a]An interaction is defined as a minimum distance between beads in the residues to be < 4 Å.

Table S4. Percentage of the total simulation time in which residues are in contact with the Lys lipid inhibitors that are bound in the main channel of GlyT2. Only interactions that occur for >30% of the total simulation time are reported.[a]

Residue	Region	C16ω7 L-Lys	C14ω5 L-Lys	C16ω7 D-Lys	C14ω5 D-Lys
V214	TM1	68.51	70.64	-	-
L436	TM5	57.39	79.69	31.03	53.73
R439	TM5	70.91	82.82	44.07	73.57
G440	TM5	-	-	-	48.74
L443	TM5	53.79	59.45	42.21	80.83
V519	TM7	34.95	61.25	-	-
I520	TM7	58.39	61.72	-	-
S522	TM7	84.75	59.92	-	-
V523	TM7	88.48	85.29	-	54.66
F526	TM7	58.79	69.57	97.6	99.73
M527	TM7	48.40	19.57	61.52	34.35
I545	EL4	78.70	-	-	-
V549	EL4	58.59	36.09	-	-
Y550	EL4	99.53	99.27	33.56	36.35
A553	EL4	84.75	59.92	45.54	64.98
L554	EL4	32.82	-	-	51.4
R556	EL4	-	33.89	97.4	73.9
L557	EL4	81.09	75.50	49.93	67.18
W563	TM8	48.40	36.09	-	-
F567	TM8	92.01	91.21	32.96	-
M570	TM8	-	30.29	-	-

[a]An interaction is defined as a minimum distance between beads in the residues to be < 4 Å.

Table S5. Percentage of the total simulation time in which residues are in contact with the Lys lipid inhibitors that are bound in the main channel of GlyT2. Only interactions that occur for >30% of the total simulation time are reported.[a]

Residue	Region	C18ω3 L-Lys	C18ω3 D-Lys	C16ω3 L-Lys	C16ω3 D-Lys
V214	TM1	75.10	32.73	73.30	-
W215	TM1	57.26	-	48.87	-
L436	TM5	86.55	77.73	75.03	61.32
R439	TM5	98.54	38.47	99.47	90.28
G440	TM5	57.86	-	68.97	83.42
L443	TM5	94.47	49.80	70.64	98.20
V519	TM7	66.84	44.33	67.24	-
I520	TM7	62.12	47.53	74.23	83.75
S522	TM7	72.97	-	66.44	85.35
V523	TM7	72.70	63.80	53.26	93.74
F526	TM7	81.82	92.87	55.19	82.42
M527	TM7	-	33.33	-	-
I545	EL4	41.41	-	-	-
V549	EL4	-	-	-	66.11
Y550	EL4	96.54	85.33	90.61	95.94
A553	EL4	-	82.33	-	75.30
L554	EL4	43.21	60.60	30.83	34.29
R556	EL4	30.89	87.60	-	-
L557	EL4	82.49	65.33	61.85	82.69
W563	TM8	40.61	31.27	63.38	69.71
F567	TM8	94.41	84.07	99.13	80.49
M570	TM8	58.79	31.53	55.33	-

[a]An interaction is defined as a minimum distance between beads in the residues to be < 4 Å.

Table S6. Hydrogen bonding interactions[a] occurring between the transmembrane helices in GlyT2 when no bioactive lipid is bound (control) or a bioactive lipid is bound for >30% of the total simulation time.

	Donor	Acceptor	Control	C18ω9 D-Trp	C18ω9 L-Trp	C18ω9 D-Lys	C18ω9 L-Lys	C16ω7 L-Lys	C16ω7 D-Lys	C14ω5 L-Lys	C14ω5 D-Lys	C18ω5 L-Lys	C18ω5 D-Lys	C16ω3 L-Lys	C16ω3 D-Lys
**TM5-TM1**	**Y423(OH)**	**D199(Oδ2)**	31%	-	-	-	33%	-	30%	-	-	-	-	31%	-
**TM1-TM7**	**N213(NδH)**	**S513(Oγ)**	38%	-	-	31%	-	-	-	-	-	-	36%	40%	-
**TM2-TM5**	**G227(NH)**	**I454(O)**	47%	-	-	-	-	-	-	-	-	-	-	-	-
**TM6-TM2**	**S477(OγH)**	**Y233(OH)**	-	50%	42%	42%	-	41%	48%	42%	39%	56%	43%	-	55%
**TM6-TM2**	**S489(OγH)**	**V249(O)**	33%	32%	-	32%	43%	41%	-	46%	42%	31%	-	-	46%
**TM2-TM7**	**Q253(NϵH)**	**N496(O)**	34%	31%	31%	32%	33%	33%	-	42%	37%	-	-	-	33%
**TM2-TM7**	**Y233(OH)**	**S513(Oγ)**	-	35%	-	-	-	-	-	-	-	-	-	-	-
**TM3-EL2**	**Y297(OH)**	**S371(Oγ)**	-	37%	-	42%	-	-	43%	-	48%	47%	36%	39%	-
**TM3-TM10**	**Y286(OH)**	**D633(Oδ2)**	34%	-	30%	-	30%	35%	34%	33%	33%	34%	-	34%	-
**TM3-TM10**	**Y286(OH)**	**D633(Oδ1)**	31%	34%	31%	32%	30%	31%	32%	33%	33%	-	-	30%	-
**TM3-TM12**	**A268(NH)**	**V751(O)**	33%	-	-	-	-	-	-	-	-	-	-	-	-
**TM5-TM7**	**Y430(OH)**	**T512(Oγ1)**	50%	46%	-	37%	-	36%	51%	30%	49%	-	-	-	48%
**TM10-EL2**	**Y627(OH)**	**S383(O)**	56%	41%	42%	33%	-	-	36%	-	32%	-	-	-	31%
**TM10-TM11**	**Y654(OH)**	**A682(O)**	-	39%	-	-	46%	57%	30%	59%	-	30%	-	-	59%
**TM12-TM10**	**Y710(OH)**	**Q630(Oϵ1)**	50%	-	-	-	-	-	-	-	-	-	-	-	-
**TM12-TM10**	**Y710(OH)**	**Y627(O)**	-	45%	-	-	-	-	-	-	-	-	-	-	-

[a]A hydrogen bond is defined as a heavy atom distance of < 3.0 Å and an angle of <20° from planar.

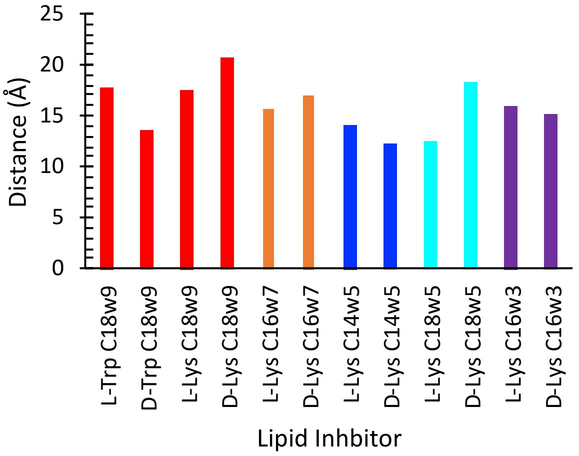

Figure S1. Average intermolecular distance (Å) from the end of the tail to the stereocenter of lipid inhibitors.

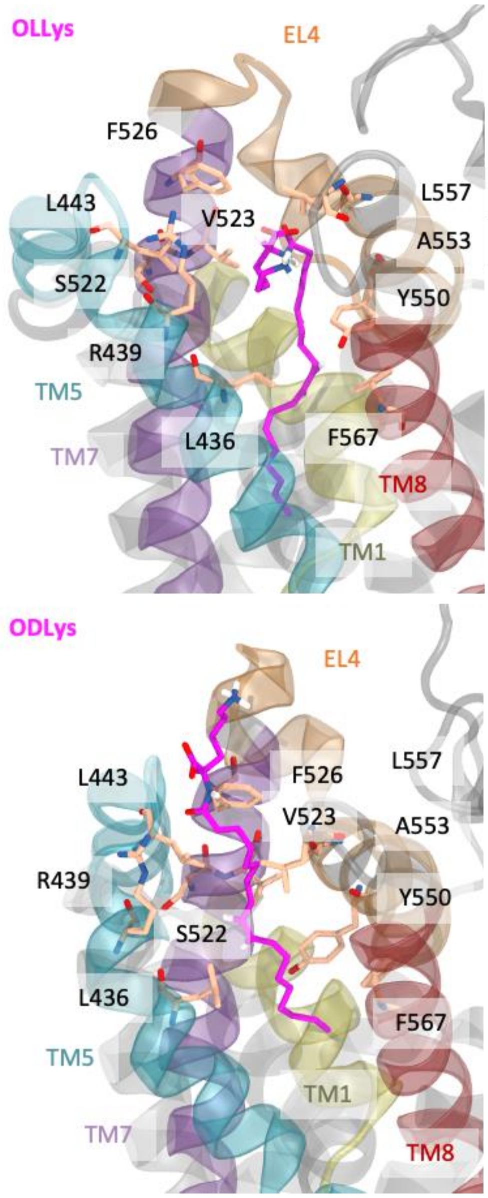

Figure S2. Positioning of key amino acids around the C18ω9 Lys lipid inhibitors bound in the extracellular allosteric binding pocket of GlyT2.

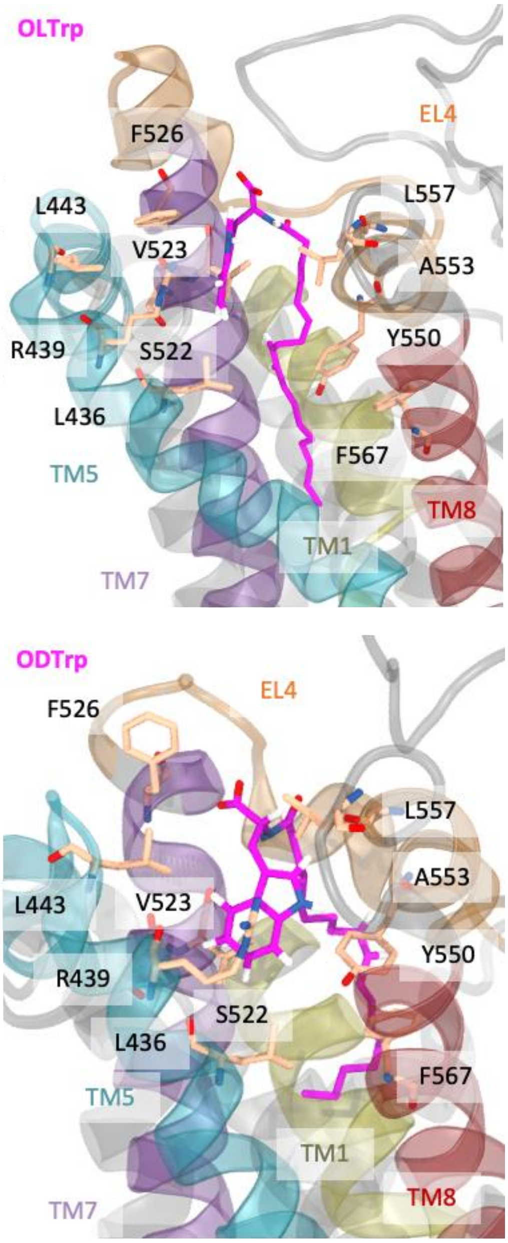

Figure S3. Positioning of key amino acids around the C18ω9 Trp lipid inhibitors bound in the extracellular allosteric binding pocket of GlyT2.

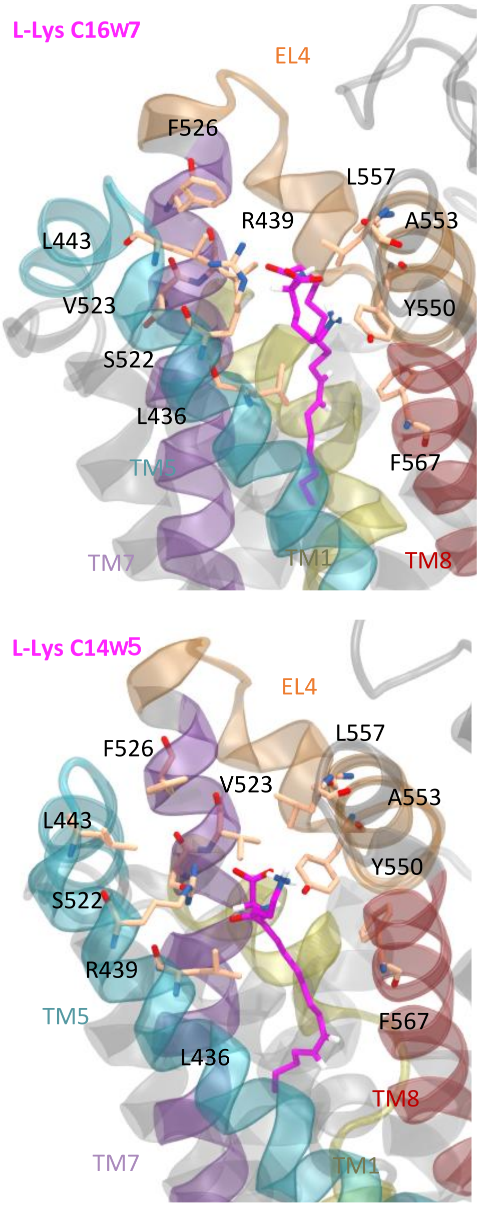

Figure S4. Positioning of key amino acids around the acyl lysine lipid inhibitors with varying tail lengths bound in the extracellular allosteric binding pocket of GlyT2.

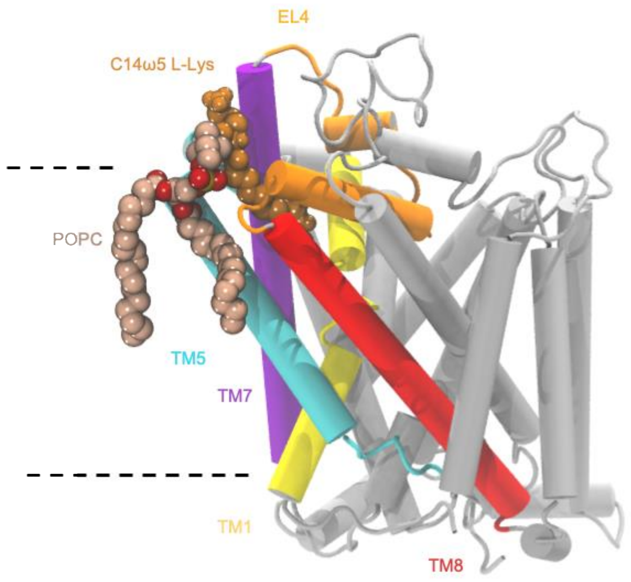

Figure S5. Interactions between C14ω5 L-Lys headgroup and membrane POPC.

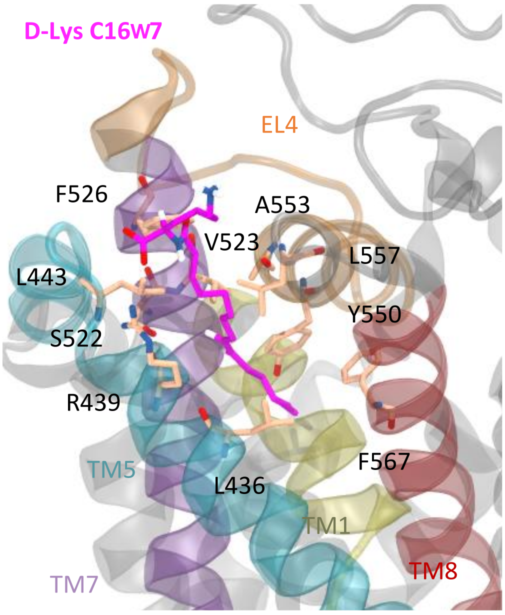

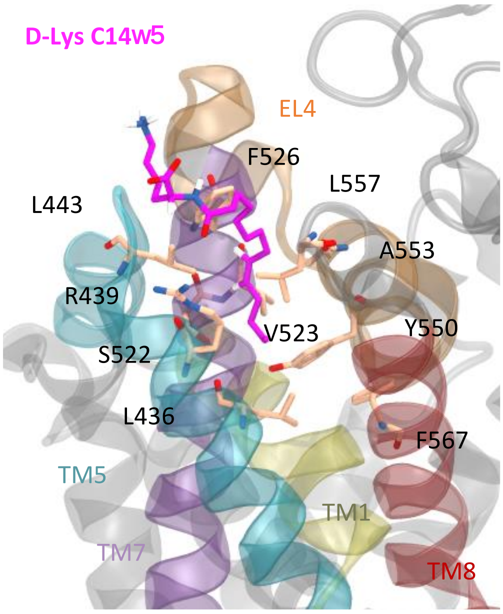

Figure S6. Positioning of key amino acids around the acyl lysine lipid inhibitors with varying tail lengths bound in the extracellular allosteric binding pocket of GlyT2.

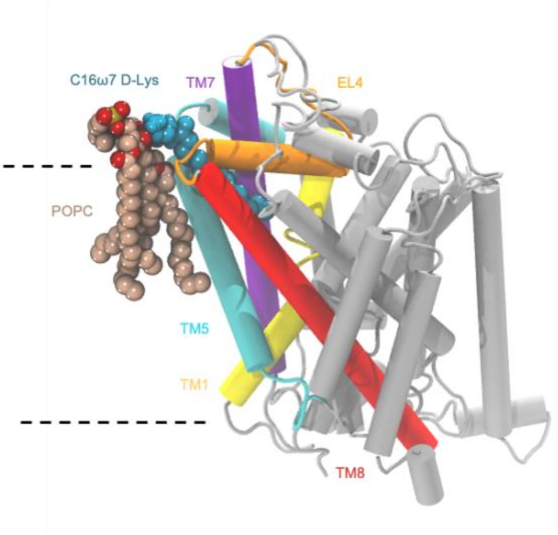

Figure S7. Interactions between C14ω5 L-Lys headgroup and membrane POPC.

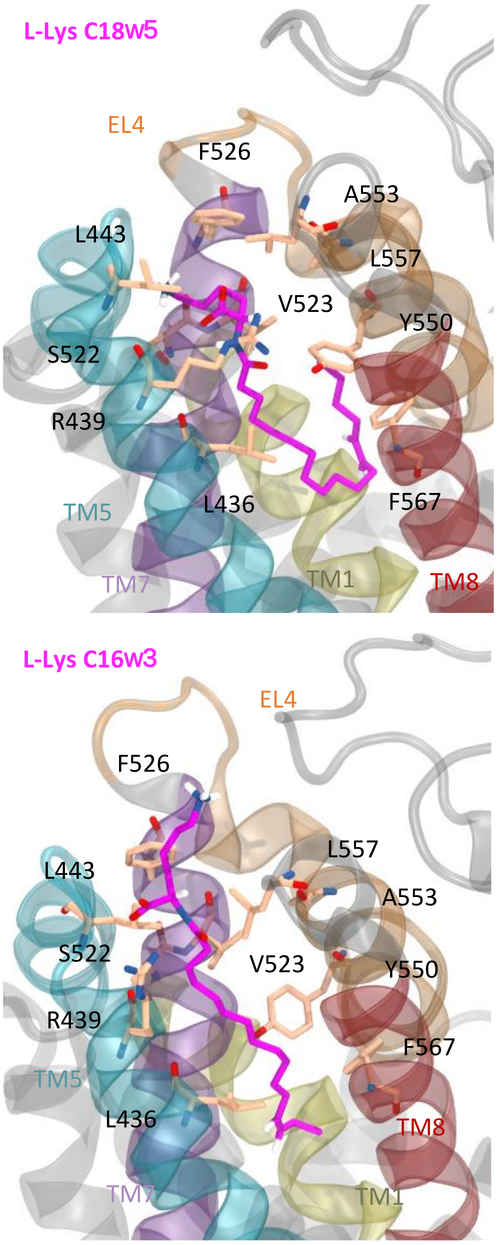

Figure S8. Positioning of key amino acids around the acyl lysine lipid inhibitors with varying double bond position bound in the extracellular allosteric binding pocket of GlyT2.

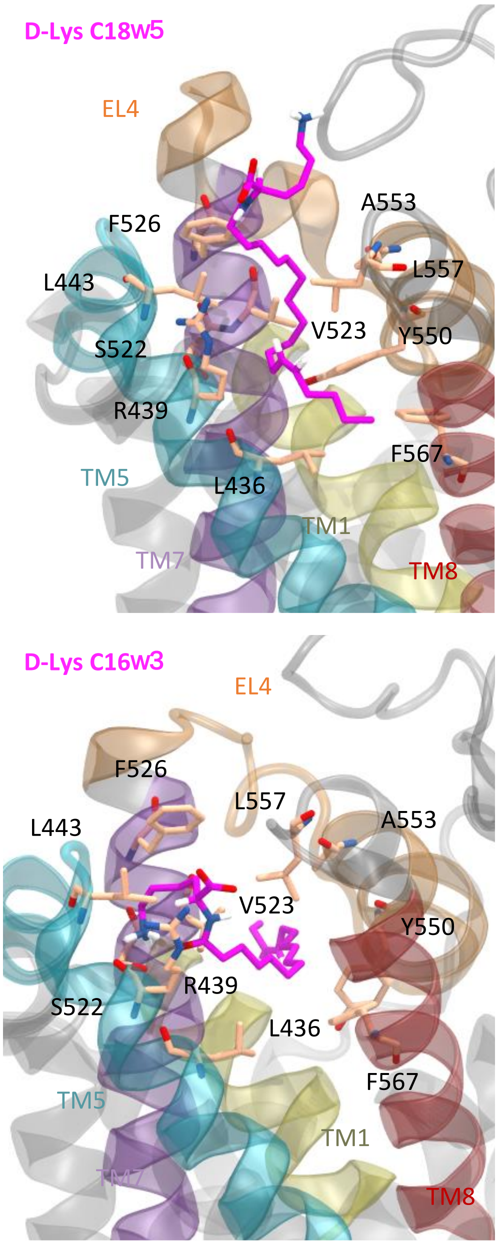

Figure S9. Positioning of key amino acids around the acyl lysine lipid inhibitors with varying double bond position bound in the extracellular allosteric binding pocket of GlyT2.

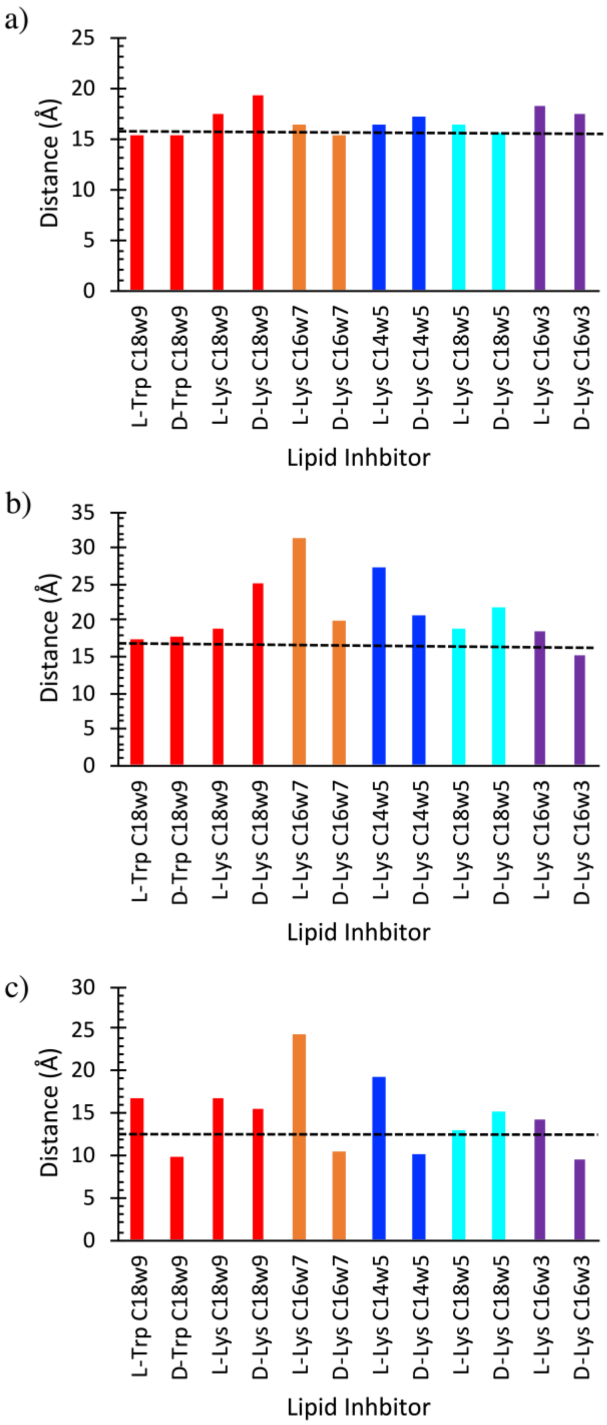

Figure S10. Average distance (Å) between a) EL2 (D329) and EL4 (Q541), b) EL2 (D329) and EL6 (Y705) and c) EL4 (Q541) and EL6 (Y705) over the total simulation time when the lipid inhibitors are bound in the extracellular allosteric binding site of GlyT2. Average distance in control simulation is indicated by a dashed line.

Synthetic Data

*Methyl(2S)-6-(tert-butoxycarbonylamino)-2-[[(Z)-octadec-13-enoyl]amino]hexanoate. (**C18ω5-L-Lysine-BOC-Me**)*

^{1}H NMR (500 MHz, DMSO-d_6): δ 8.10 (d, *J* = 7.5 Hz, 1H), 6.74 (t, *J* = 5.5 Hz, 1H), 5.35 - 5.25 (m, 2H), 4.20 - 4.10 (m, 1H), 3.60 (s, 3H), 2.90 - 2.80 (m, 2H), 2.08 (t, *J* = 7.5 Hz, 2H), 2.00 - 1.95 (m, 4H), 1.66 - 1.60 (m, 1H), 1.58-1.50 (m, 1H), 1.47 - 1.40 (m, 2H), 1.40 - 1.10 (m, 33H), 0.85 (t, *J* = 7.0 Hz, 3H). ^{13}C NMR (125 MHz, DMSO-d_6): δ 172.82, 172.40, 155.55, 129.61, 129.57, 77.28, 51.76, 51.65, 34.90, 31.35, 30.54, 29.08, 29.03, 29.00, 28.98, 28.96(2C), 28.85, 28.77, 28.57, 28.54, 28.24(3C), 26.55, 26.30, 25.20, 22.71 21.70, 13.79. HRMS (ESI) *m/z* $[M+H]^+$ calcd for $C_{30}H_{57}N_2O_5$, 525.4262; found, 525.4262.

*Methyl(2R)-6-(tert-butoxycarbonylamino)-2-[[(Z)-octadec-13-enoyl]amino]hexanoate (**C18ω5-D-Lysine-BOC-Me**)*

^{1}H NMR (500 MHz, $CDCl_3$), 6.03 (d, *J* = 5.5 Hz, 1H), 5.40 - 5.30 (m, 2H), 4.60 -4.50 (m, 2H), 3.74 (s, 3H), 3.07 (q, *J* = 7.5 Hz, 2H), 2.15 (t, *J* = 7.5 Hz, 2H), 2.10 - 2.00 (m, 4H), 1.80 - 1.70 (m, 1H), 1.75 - 1.60 (m, 3H), 1.55 - 1.45 (m, 2H), 1.44 (s, 9H), 1.40 - 1.20 (m, 22H), 0.89 (t, *J* = 7.5 Hz, 3H). ^{13}C NMR (125 MHz, $CDCl_3$): δ 173.24, 173.13, 156.25, 130.11, 129.89, 79.88, 52.50, 51.93, 36.70, 32.23, 31.96, 29.92, 29.75, 29.69, 29.64(2C), 29.48(2C), 29.46(2C), 29.41, 28.57(3C), 27.35, 27.05, 25.73, 22.51, 22.49, 14.14. HRMS (ESI) *m/z* $[M+H]^+$ calcd for $C_{30}H_{57}N_2O_5$, 525.4262; found, 525.4260.

*methyl (2S)-6-(tert-butoxycarbonylamino)-2-[[(Z)-hexadec-13-enoyl]amino]hexanoate (**C16ω3-L-Lys-BOC-Me**)*

^{1}H NMR (500 MHz, DMSO-d_6): δ 8.09 (d, *J* = 7.5 Hz, 1H), 6.74 (t, *J* = 5.0 Hz, 1H), 5.40 - 5.25 (m, 2H), 4.20 - 4.15 (m, 1H), 3.59 (s, 3H), 2.87 (q, *J* = 6.5 Hz, 2H), 2.09 (t, *J* = 7.5 Hz, 2H), 2.00 - 1.90 (m, 4H), 1.70 - 1.60 (m, 1H), 1.60 - 1.50 (m, 1H), 1.45 (p, *J* = 7.0 Hz, 2H), 1.40 - 1.10 (m, 29H), 0.90 (t, *J* = 7.0 Hz, 3H). ^{13}C NMR (125 MHz, DMSO-d_6): δ 172.83, 172.42, 155.55, 131.26, 129.01, 77.31, 51.76, 51.65, 34.90, 30.53, 29.11, 29.03, 29.00, 28.97, 28.94, 28.87, 28.75, 28.57 (2C), 58.52, 28.25 (3C), 26.47, 25.20, 22.71, 20.00, 14.23. HRMS (ESI) *m/z* $[M+H]^+$ calcd for $C_{28}H_{53}N_2O_5$, 497.3949; found, 497.3945.

*Methyl (2R)-6-(tert-butoxycarbonylamino)-2-[[(Z)-hexadec-13-enoyl]amino]hexanoate. (**C16ω3-D-Lys-BOC-Me**)*

^{1}H NMR (500 MHz, $CDCl_3$): 6.10 - 6.05 (m, 1H), 5.40 - 5.30 (m, 2H), 4.60 - 4.55 (m, 2H), 3.72 (s, 3H), 3.10 - 3.00 (m, 2H), 2.20 (t, *J* = 7.5 Hz, 2H), 2.05 - 1.95 (m, 4H), 1.85 - 1.70 (m, 1H), 1.70 - 1.55 (m, 3H), 1.50 - 1.45 (m, 2H), 1.41 (m, 9H), 1.35 - 1.20 (m, 18H), 0.93 (t, *J* = 7.0 Hz, 3H). ^{13}C NMR (125 MHz, $CDCl_3$): δ 173.23, 173.15, 156.22, 131.61, 129.45, 79.24, 52.46, 51.91, 40.14, 36.68, 32.18, 29.90, 29.72 (2C), 29.66, 29.61, 29.45, 29.41, 29.38, 28.54 (3C), 27.21, 25.71, 22.50, 20.62, 14.51. HRMS (ESI) *m/z* $[M+H]^+$ calcd for $C_{28}H_{53}N_2O_5$, 497.3949; found, 497.3947.

*Methyl (2S)-6-(tert-butoxycarbonylamino)-2-[[(Z)-hexadec-9-enoyl]amino]hexanoate. (**C16ω7-L-Lys-BOC-Me**)*

^{1}H NMR (500 MHz, DMSO-d_6): δ 8.09 (d, *J* = 7.5 Hz, 1H), 6.75 (t, *J* = 5.5 Hz, 1H), 5.35 - 5.25 (m, 2H), 4.20 - 4.15 (m, 1H), 3.59 (s, 3H), 2.90 - 2.80 (m, 2H), 2.09 (t, *J* = 7.5 Hz, 2H), 2.00 - 1.95 (m, 4H), 1.65-

1.60 (m, 1H), 1.60 - 1.50 (m, 1H), 1.48-1.42 (m, 2H), 1.40 - 1.20 (m, 29H), 0.84 (t, *J* = 7.0 Hz, 3H). ^{13}C NMR (125 MHz, $CDCl_3$): δ 173.24, 173.13, 156.26, 130.12, 129.89, 79.30, 52.50, 51.93, 40.17, 36.70, 32.23, 31.92, 29.87, 29.85, 29.75, 29.53, 29.38, 29.29, 29.23, 29.12, 28.55 (3C), 27.36, 27.32, 25.72, 22.80, 22.51, 14.25. HRMS (ESI) *m/z* $[M+H]^+$ calcd for $C_{28}H_{53}N_2O_5$, 497.3949; found, 497.3955.

*Methyl (2R)-6-(tert-butoxycarbonylamino)-2-[[(Z)-hexadec-9-enoyl]amino]hexanoate. (**C16ω7-D-Lys-BOC-Me**)*

^{1}H NMR (500 MHz, $CDCl_3$): 6.05 (d, *J* = 7.0 Hz, 1H), 5.40 - 5.30 (m, 2H), 4.65 -4.50 (m, 2H), 3.73 (s, 3H), 3.09 (q, *J* = 7.5 Hz, 2H), 2.21 (t, *J* = 7.5 Hz, 2H), 2.05 - 1.95 (m, 4H), 1.90 - 1.80 (m, 1H), 1.75 - 1.55 (m, 3H), 1.55 - 1.40 (m, 11H), 1.40 - 1.20 (m, 18H), 0.89 (t, *J* = 7.0 Hz, 3H). ^{13}C NMR (125 MHz, $CDCl_3$): δ 173.24, 173.13, 156.26, 130.11, 129.89, 79.36, 52.50, 51.93, 40.14, 36.70, 32.23, 31.92, 29.87, 29.85, 29.75, 29.38, 29.29, 29.22, 29.12, 28.56 (3C), 27.36, 27.31, 25.71, 22.79, 22.51, 14.51. HRMS (ESI) *m/z* $[M+H]^+$ calcd for $C_{28}H_{53}N_2O_5$, 497.3949; found, 497.3952.

*Methyl (2S)-6-(tert-butoxycarbonylamino)-2-[[(Z)-tetradec-9-enoyl]amino]hexanoate (**C14ω5-L-Lys-BOC-Me**)*

^{1}H NMR (500 MHz, $CDCl_3$): δ 6.06 (d, *J* = 7.0 Hz, 1H), 5.35 - 5.30 (m, 2H), 4.60 - 4.50 (m, 2H), 3.74 (s, 3H), 3.09 (q, *J* = 7.5 Hz, 2H), 2.22 (t, *J* = 7.5 Hz, 2H), 2.05-1.98 (m, 4H), 1.90 -1.80 (m, 1H), 1.66 - 1.60 (m, 3H), 1.47 - 1.40 (m, 11H), 1.35 - 1.20 (m, 14H), 0.85 (t, *J* = 7.0 Hz, 3H). ^{13}C NMR (125 MHz, $CDCl_3$): δ 173.22, 173.21, 156.27, 130.03, 129.87, 79.31, 52.47, 51.92, 36.64, 32.18, 32.06, 32.03, 29.81, 29.76, 29.71, 29.33, 29.24, 28.52(3C), 27.26, 27.02, 25.69, 22.49, 22.44, 14.10. HRMS (ESI) *m/z* $[M+H]^+$ calcd for $C_{26}H_{49}N_2O_5$, 469.3636, found, 469.3639.

*Methyl (2R)-6-(tert-butoxycarbonylamino)-2-[[(Z)-tetradec-9-enoyl]amino]hexanoate (**C14ω5-D-Lys-BOC-Me**)*

^{1}H NMR (500 MHz, $CDCl_3$): δ 6.08 (d, *J* = 7.0 Hz, 1H), 5.35 - 5.30 (m, 2H), 4.60 - 4.55 (m, 2H), 3.75 (s, 3H), 3.09 (q, *J* = 7.5 Hz, 2H), 2.21 (t, *J* = 7.5Hz, 2H), 2.00 - 1.95 (m, 4H), 1.90 - 1.80 (m, 1H), 1.64 - 1.45 (m, 3H), 1.47 - 1.40 (m, 11H), 1.40 - 1.20 (m, 14H), 0.84 (t, *J* = 7.0 Hz, 3H). ^{13}C NMR (125 MHz, $CDCl_3$): δ 173.22(2C), 156.27, 130.03, 129.87, 79.28, 52.46, 51.96, 36.63, 32.17, 32.06, 29.81, 29.71, 29.33(2C), 29.24(2C), 28.52(3C), 27.26, 27.02, 25.69, 22.49, 22.44, 14.10. HRMS (ESI) *m/z* $[M+H]^+$ calcd for $C_{26}H_{49}N_2O_5$, 469.3636, found, 469.3638.

*(2S)-6-(tert-butoxycarbonylamino)-2-[[(Z)-octadec-13-enoyl]amino]hexanoic acid (**C18ω5-L-Lys-BOC**)*

^{1}H NMR (500 MHz, DMSO-d_6): δ 7.95 (d, *J* = 7.5 Hz, 1H), 6.74 (t, *J* = 5.5 Hz, 1H), 5.40 - 5.30 (m, 2H), 4.15 - 4.10 (m, 1H), 2.90 - 2.80 (m, 2H), 2.15 - 2.05 (m, 2H), 2.00 - 1.90 (m, 4H), 1.70 - 1.60 (m, 1H), 1.60 - 1.40 (m, 3H), 1.40 - 1.15 (m, 33H), 0.84 (t, *J* = 7.0 Hz, 3H). ^{13}C NMR (125 MHz, DMSO-d_6): δ 173.84, 172.24, 155.53, 129.61, 129.56, 77.28, 51.65, 35.03, 31.36, 30.72, 29.08, 29.01, 28.97, 28.86, 28.79, 28.57 (2C), 28.25 (3C), 26.55, 26.30, 25.26, 22.81, 21.71, 20.75, 13.80. HRMS (ESI) *m/z* $[M+H]^+$ calcd for $C_{29}H_{55}N_2O_5$, 511.4105 found, 511.4102.

*(2R)-6-(tert-butoxycarbonylamino)-2-[[(Z)-octadec-13-enoyl]amino]hexanoic acid (**C18ω5-D-Lys-BOC**)*

^{1}H NMR (500 MHz, DMSO-d_6): δ 7.96 (d, *J* = 8.0 Hz, 1H), 6.74 (t, *J* = 5.5Hz, 1H), 5.45 - 5.30 (m, 2H), 4.15 - 4.10 (m, 1H), 2.86 (q, *J* = 6.0 Hz, 2H), 2.10 - 2.05 (m, 2H), 2.00 - 1.90 (m, 4H), 1.70 - 1.60 (m, 1H), 1.55 - 1.40 (m, 3H), 1.40 - 1.10 (m, 33H), 0.84 (t, *J* = 7.0Hz, 3H). ^{13}C NMR (125 MHz, DMSO-d_6): δ 173.87, 172.31, 155.56, 129.63, 129.59, 77.32, 51.67, 35.05, 31.36, 30.72, 29.08 (2C), 29.01, 28.97, 28.88, 28.79, 28.60 (2C), 28.27 (3C), 26.58, 26.30, 25.26, 22.81, 21.73, 13.80. HRMS (ESI) *m/z* $[M+H]^+$ calcd for $C_{29}H_{55}N_2O_5$, 511.4105; found, 511.4104.

*(2S)-6-(tert-butoxycarbonylamino)-2-[[(Z)-hexadec-13-enoyl]amino]hexanoic acid (**C16ω3-L-Lys-BOC**)*

^{1}H NMR (500 MHz, DMSO-d_6): δ 7.95 (d, *J* = 8.0 Hz, 1H), 6.73 (t, *J* = 5.5 Hz, 1H), 5.35 - 5.25 (m, 2H), 4.15 - 4.10 (m, 1H), 2.90 - 2.80 (m, 2H), 2.15 - 2.05 (m, 2H), 2.00 - 1.95 (m, 4H), 1.65-1.60 (m, 1H), 1.55 - 1.40 (m, 3H), 1.40 - 1.20 (m, 29H), 0.90 (t, *J* = 7.0 Hz, 3H). ^{13}C NMR (125 MHz, DMSO-d_6): δ 173.88, 172.32, 155.16, 131.29, 129.04, 77.35, 51.70, 35.06, 30.75, 29.14, 29.03, 29.02 (2C), 28.98, 28.90, 28.81, 28.61 (2C), 28.28 (3C), 26.47, 25.29, 22.84, 20.03, 14.23. HRMS (ESI) *m/z* $[M+H]^+$ calcd for $C_{27}H_{51}N_2O_5$, 483.3792; found, 483.3789.

*(2R)-6-(tert-butoxycarbonylamino)-2-[[(Z)-hexadec-13-enoyl]amino]hexanoic acid (**C16ω3-D-Lys-BOC**)*

^{1}H NMR (500 MHz, DMSO-d_6): δ 7.94 (d, *J* = 8.0 Hz, 1H), 6.74 (t, *J* = 7.0 Hz, 1H), 5.35 - 5.25 (m, 2H), 4.15 - 4.10 (m, 1H), 2.90 - 2.80 (m, 2H), 2.10 - 2.05 (m, 2H), 2.00 - 1.90 (m, 4H), 1.70 - 1.60 (m, 1H), 1.55 - 1.40 (m, 3H), 1.40 - 1.20 (m, 29H), 0.90 (t, *J* = 7.0 Hz, 3H). ^{13}C NMR (500 MHz, DMSO d_6): δ 173.85, 172.24, 155.53, 131.26, 129.01, 77.29, 51.67, 35.03, 30.74, 29.11, 29.00, 28.99 (2C), 28.95, 28.87, 28.78, 28.58 (2C), 28.25 (3C), 26.47, 25.29, 22.82, 20.00, 13.97. HRMS (ESI) *m/z* $[M+H]^+$ calcd for $C_{27}H_{51}N_2O_5$, 483.3792; found, 483.3790.

*(2S)-6-(tert-butoxycarbonylamino)-2-[[(Z)-hexadec-9-enoyl]amino]hexanoic acid (**C16ω7-L-Lys-BOC**)*

^{1}H NMR (500 MHz, DMSO-d_6): δ 7.94 (d, *J* = 8.0 Hz, 1H), 6.74 (t, *J* = 5.0 Hz, 1H), 5.35 - 5.25 (m, 2H), 4.15 - 4.10 (m, 1H), 2.90 - 2.80 (m, 2H), 2.15 - 2.05 (m, 2H), 2.00 - 1.90 (m, 4H), 1.65 - 1.60 (m, 1H), 1.55 - 1.40 (m, 3H), 1.35 - 1.15 (m, 29H), 0.84 (t, *J* = 7.0 Hz, 3H). ^{13}C NMR (125 MHz, DMSO-d_6): δ 173.82, 172.20, 155.55, 129.63 (2C), 77.30, 51.72, 35.05, 31.13 (2C), 30.77, 29.11, 29.09 (2C), 28.67, 28.56 (2C), 28.27 (3C), 26.60 (2C), 25.26, 22.09, 18.22, 13.95. HRMS (ESI) *m/z* $[M+H]^+$ calcd for $C_{27}H_{51}N_2O_5$, 483.3792; found, 483.3794.

*(2R)-6-(tert-butoxycarbonylamino)-2-[[(Z)-hexadec-9-enoyl]amino]hexanoic acid (**C16ω7-D-Lyd-BOC**)*

^{1}H NMR (500 MHz, DMSO-d_6): δ 7.92 (d, *J* = 7.5 Hz, 1H), 6.74 (t, *J* = 5.0 Hz, 1H), 5.35 - 5.25 (m, 2H), 4.15 - 4.10 (m, 1H), 2.87 (q, *J* = 7.0 Hz, 2H), 2.15 - 2.05 (m, 2H), 2.00 - 1.90 (m, 4H), 1.70 - 1.60 (m, 1H), 1.55 - 1.40 (m, 3H), 1.40 - 1.15 (m, 29H), 0.84 (t, *J* = 7.0 Hz, 3H). ^{13}C NMR (125 MHz, DMSO-d_6): δ 173.89, 172.29, 155.59, 129.67 (2C), 77.36, 51.78, 35.08, 31.16 (2C), 30.80, 29.14, 29.11 (2C), 28.68, 28.60, 28.58, 28.30 (3C), 26.63 (2C), 25.29, 22.82, 22.11, 13.97. HRMS (ESI) *m/z* $[M+H]^+$ calcd for $C_{27}H_{51}N_2O_5$, 483.3792; found, 483.3793.

*(2S)-6-(tert-butoxycarbonylamino)-2-[[(Z)-tetradec-9-enoyl]amino]hexanoic acid (**C14ω5-L-Lys-BOC**)*

^{1}H NMR (500 MHz, DMSO-d_6): δ 7.95 (d, *J* = 8.0Hz, 1H), 6.75 - 6.70 (m, 1H), 5.35 - 5.25 (m, 2H), 4.15 - 4.10 (m, 1H), 2.90 - 2.80 (m, 2H), 2.10 - 2.05 (m, 2H), 2.00 - 1.95 (m, 4H), 1.65 - 1.60 (m, 1H), 1.55 - 1.40 (m, 3H), 1.40 - 1.20 (m, 25H), 0.85 (t, *J* = 7.0Hz, 3H). ^{13}C NMR (125 MHz, DMSO-d_6): δ 173.85, 172.24, 155.53, 129.61, 129.57, 77.29, 51.77, 35.03, 31.35, 30.73, 29.10, 29.65, 28.57(2C), 28.55, 28.25 (3C), 26.59, 26.30, 25.25, 22.82, 21.70, 13.10. HRMS (ESI) m/z [M+H]+ calcd for $C_{25}H_{47}N_2O_5$, 455.3480 found, 455.3484.

*(2R)-6-(tert-butoxycarbonylamino)-2-[[(Z)-tetradec-9-enoyl]amino]hexanoic acid (**C14ω5-D-Lys-BOC**)*

^{1}H NMR (500 MHz, DMSO-d_6): δ 7.95 (d, *J* = 8.0 Hz, 1H), 6.75-6.70 (m, 1H), 5.40 - 5.30 (m, 2H), 4.15 - 4.10 (m, 1H), 2.90 - 2.80 (m, 2H), 2.10 - 2.06 (m, 2H), 2.00 - 1.95 (m, 4H), 1.65 - 1.60 (m, 1H), 1.55 - 1.45 (m, 3H), 1.40 - 1.20 (m, 25H), 0.85 (t, *J* = 7.5 Hz, 3H). ^{13}C NMR (125 MHz, DMSO-d_6): δ 173.86, 172.24, 156.53, 129.61, 129.57, 77.30, 51.67, 35.03, 31.36, 30.73, 29.01, 28.65, 28.57 (2C), 28.55, 28.26 (3C), 26.59, 26.30, 25.69, 22.49, 22.44, 14.10. HRMS (ESI) *m/z* $[M+H]^+$ calcd for $C_{25}H_{47}N_2O_5$, 455.3480, found, 455.3477.

*(2S)-6-amino-2-[[(Z)-octadec-13-enoyl]amino]hexanoic acid.HCl (**C18ω5-L-Lys**)*

^{1}H NMR (500 MHz, DMSO-d_6): δ 8.04 (d, *J* = 8.0 Hz, 1H), 7.89 (s, 3H), 5.35 - 5.30 (m, 2H), 4.15 - 4.10 (m, 1H), 2.80 - 2.70 (m, 2H), 2.09 (t, *J* = 7.0 Hz, 2H), 2.00 - 1.90 (m, 4H), 1.70 - 1.65 (m, 1H), 1.60 - 1.40 (m, 5H), 1.40 - 1.20 (m, 22H), 0.84 (t, *J* = 7.0 Hz, 3H). ^{13}C NMR (125 MHz, DMSO-d_6): δ 173.71, 172.35, 129.62, 129.58, 51.49, 38.43, 35.03, 31.36, 30.37, 29.09, 29.01 (2C), 28.97, 28.86, 28.82, 28.64, 28.58, 28.56, 26.47, 26.31, 25.25, 22.44, 21.71, 13.81. HRMS (ESI) *m/z* $[M+H]^+$ calcd for $C_{24}H_{47}N_2O_3$, 411.3581; found,411.3584.

*(2R)-6-amino-2-[[(Z)-octadec-13-enoyl]amino]hexanoic acid.HCl (**C18ω5-D-Lys**)*

^{1}H NMR (500 MHz, DMSO-d_6): δ 8.05 (d, *J* = 8.0 Hz, 1H), 7.91 (s, 3H), 5.35 -5.25 (m, 2H), 4.16 - 4.10 (m, 1H), 2.80 - 2.70 (m, 2H), 2.09 (t, *J* = 7.0 Hz, 2H), 2.00 - 1.90 (m, 4H), 1.70 - 1.60 (m, 1H), 1.60 - 1.40 (m, 5H), 1.40 - 1.20 (m, 22H), 0.84 (t, *J* = 7.0 Hz, 3H). ^{13}C NMR (125 MHz, DMSO-d_6): δ 173.73, 172.39, 129.64, 129.60, 51.54, 38.45, 35.06, 31.36, 30.37, 29.09, 29.01, 28.99 (2C), 28.88, 28.84, 28.64, 28.58, 28.56, 26.47, 26.31, 25.25, 22.44, 21.73, 13.83. HRMS (ESI) *m/z* $[M+H]^+$ calcd for $C_{24}H_{47}N_2O_3$, 411.3581; found,411.3583.

*(2S)-6-amino-2-[[(Z)-hexadec-13-enoyl]amino]hexanoic acid.HCl (**C16ω3-L-Lys**)*

^{1}H NMR (500 MHz, DMSO d_6): δ 8.05 (d, *J* = 8.0 Hz, 1H), 7.90 (s, 3H), 5.35 - 5.25 (m, 2H), 4.16 - 4.10 (m, 1H), 2.80 - 2.70 (m, 2H), 2.10 (t, *J* = 7.5 Hz, 2H), 2.00 - 1.90 (m, 4H), 1.70 - 1.65 (m, 1H), 1.60 - 1.45 (m, 5H), 1.40 - 1.20 (m, 18H), 0.90 (t, *J* = 7.0 Hz, 3H). ^{13}C NMR (125 MHz, DMSO-d_6): δ 173.73, 172.35, 131.27, 129.01, 51.49, 36.66, 35.03, 30.36, 29.11, 29.01(2C), 28.96(2C), 28.88, 28.81, 28.62, 28.59, 26.47, 25.24, 22.42, 20.00, 14.24. HRMS (ESI) *m/z* $[M+H]^+$ calcd for $C_{22}H_{43}N_2O_3$, 383.3268; found, 383.3268.

(2R)-6-amino-2-[[(Z)-hexadec-13-enoyl]amino]hexanoic acid.HCl ***(C16ω3-D-Lys)***

^{1}H NMR (500 MHz, DMSO-d_6): δ 8.03 (d, *J* = 8.0 Hz, 1H), 7.88 (s, 3H), 5.35 - 5.25 (m, 2H), 4.15 - 4.10 (m, 1H), 2.72 (t, *J* = 7.0 Hz, 2H), 2.10 (t, *J* = 7.5 Hz, 2H), 2.00 - 1.90 (m, 4H), 1.68-1.60 (m, 1H), 1.60 - 1.40 (m, 5H), 1.40 - 1.20 (m, 18H), 0.90 (t, *J* = 7.0 Hz, 3H). ^{13}C NMR (125 MHz, DMSO-d_6): δ 173.73, 172.35, 131.27, 129.01, 51.49, 38.48, 35.04, 30.36, 29.11, 29.01, 28.96 (2C), 28.88, 28.81, 28.62, 28.59, 26.47 (2C), 25.24, 22.42, 20.00, 14.24. HRMS (ESI) *m/z* $[M+H]^+$ calcd for $C_{22}H_{43}N_2O_3$, 383.3268; found, 383.3263.

(2S)-6-amino-2-[[(Z)-hexadec-9-enoyl]amino]hexanoic acid.HCl ***(C16ω7-L-Lys)***

^{1}H NMR (500 MHz, DMSO-d_6): δ 8.04 (d, *J* = 7.5 Hz, 1H), 7.89 (s, 3H), 5.35–5.30 (m, 2H), 4.20 - 4.10 (m, 1H), 2.80 - 2.70 (m, 2H), 2.09 (t, *J* = 7.0 Hz, 2H), 2.00 - 1.90 (m, 4H), 1.70 - 1.60 (m, 1H), 1.60 - 1.40 (m, 5H), 1.40 - 1.20 (m, 18H), 0.84 (t, *J* = 7.0 Hz, 3H). ^{13}C NMR (125 MHz, DMSO-d_6): δ 173.73, 172.35, 129.65(2C), 51.51, 38.42, 35.06, 31.12, 30.37, 29.12, 29.08, 28.67, 28.61, 28.56, 28.26, 26.60 (2C), 26.47, 25.24, 22.43, 22.07, 13.94. HRMS (ESI) *m/z* $[M+H]^+$ calcd for $C_{22}H_{43}N_2O_3$, 383.3268; found, 383. 3266.

(2R)-6-amino-2-[[(Z)-hexadec-9-enoyl]amino]hexanoic acid.HCl ***(C16ω7-D-Lys)***

^{1}H NMR (500 MHz, DMSO-d_6): δ 8.05 (d, *J* = 7.5 Hz, 1H), 7.92 (s, 3H), 5.35–5.30 (m, 2H), 4.15 - 4.10 (m, 1H), 2.80 - 2.70 (m, 2H), 2.09 (t, *J* = 7.0 Hz, 2H), 2.00 - 1.90 (m, 4H), 1.70 - 1.65 (m, 1H), 1.60 - 1.40 (m, 5H), 1.40 - 1.20 (m, 18H), 0.84 (t, *J* = 7.0 Hz, 3H). ^{13}C NMR (125 MHz, DMSO-d_6): δ 173.71, 172.34, 129.63 (2C), 51.52, 38.40, 35.06, 31.12, 30.35, 29.12, 29.08, 28.67, 28.61, 28.56, 28.26, 26.60 (2C), 26.47, 25.24, 22.43, 22.07, 13.94. HRMS (ESI) *m/z* $[M+H]^+$ calcd for $C_{22}H_{43}N_2O_3$, 383.3268; found, 383.3265.

(2S)-6-amino-2-[[(Z)-tetradec-9-enoyl]amino]hexanoic acid.HCl ***(C14ω5-L-Lys)***

^{1}H NMR (500 MHz, DMSO-d_6): δ 8.06 (d, *J* = 7.5 Hz, 1H), 7.87 (s, 3H), 5.35 - 5.30 (m, 2H), 4.15 - 4.10 (m, 1H), 2.75 - 2.70 (m, 2H), 2.09 (t, *J* = 7.5 Hz, 2H), 2.00 - 1.90 (m, 4H), 1.70 - 1.65 (m, 1H), 1.55 - 1.40 (m, 5H), 1.40 - 1.20 (m, 14H), 0.84 (t, *J* = 7.0 Hz, 3H). ^{13}C NMR (125 MHz, DMSO-d_6): δ 173.74, 172.41, 129.62, 129.62, 51.58, 38.40, 35.06, 31.12, 30.35, 29.12, 28.67, 28.61, 28.56, 26.60, 26.49, 26.32, 25.24, 22.43, 21.72, 13.84. HRMS (ESI) *m/z* $[M+H]^+$ calcd for $C_{20}H_{39}N_2O_3$, 355.2955; found, 355.2955.

(2R)-6-amino-2-[[(Z)-tetradec-9-enoyl]amino]hexanoic acid.HCl ***(C14ω5-D-Lys)***

^{1}H NMR (500 MHz, DMSO-d_6): δ 8.06 (d, *J* = 7.5 Hz, 1H), 7.87 (s, 3H), 5.35 - 5.30 (m, 2H), 4.15 - 4.10 (m, 1H), 2.75 - 2.70 (m, 2H), 2.09 (t, *J* = 7.5 Hz, 2H), 2.00 - 1.90 (m, 4H), 1.70 – 1.65 (m, 1H), 1.60 - 1.40 (m, 5H), 1.40 - 1.20 (m, 14H), 0.84 (t, *J* = 7.0 Hz, 3H). ^{13}C NMR (125 MHz, DMSO-d_6): δ 173.71, 172.34, 129.62, 129.62, 51.52, 38.40, 35.06, 31.12, 30.35, 29.12, 28.67, 28.61, 28.56, 26.60, 26.49, 26.32, 25.24, 22.43, 21.72, 13.84. HRMS (ESI) *m/z* $[M+H]^+$ calcd for $C_{20}H_{39}N_2O_3$, 355.2955; found, 355.2957.
